# Acute Oxalate Nephropathy following Ingestion of *Averrhoa bilimbi* Juice

**DOI:** 10.1155/2014/240936

**Published:** 2014-06-04

**Authors:** Sreeja Nair, Jacob George, Sajeev Kumar, Noble Gracious

**Affiliations:** Department of Nephrology, Medical College, Thiruvananthapuram, Kerala 695011, India

## Abstract

Plant toxins are known to cause acute kidney injury in tropical countries. We report two cases of acute kidney injury with tubular oxalate deposition following ingestion of *Averrhoa bilimbi* fruit juice. Both patients had complete renal recovery though one required dialytic support.

## 1. Introduction

Plant toxins are an important cause of tropical acute kidney injury. Several plant parts are ingested for medicinal, cosmetic, and even suicidal purposes [1].* Averrhoa bilimbi* (commonly known as bilimbi, cucumber tree, tree sorrel, Irumban Puli, or Chemmeen Puli) is a plant with several suggested medicinal properties. It, however, has a high content of oxalic acid which could contribute to nephrotoxicity [2]. We describe two cases of acute oxalate nephropathy following ingestion of* Averrhoa bilimbi* juice.

## 2. Case 1

**Figure 1:**
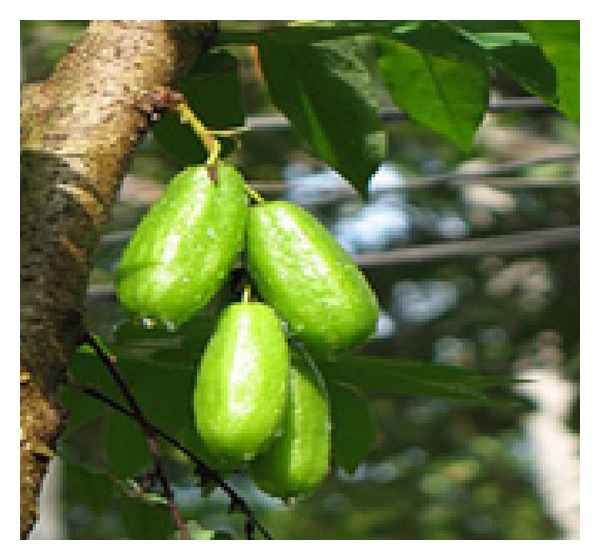
Picture of fruit of* Averrhoa bilimbi*.

A forty-five-year-old female presented to the nephrology outpatient clinic with history of bilateral pedal edema, facial puffiness, and abdominal distention of three-day duration. There was no history of oliguria, hematuria, or frothing of urine. She did not have fever, dysuria, shortness of breath, or haemoptysis. She was diagnosed with systemic hypertension 18 years back for which she was taking enalapril 2.5 mg once daily. She was detected to have dyslipidemia on routine evaluation 3 months back and was advised dietary modification and life style changes only. She was on regular follow-up and was documented to have normal renal function two months back. On probing for history of nephrotoxic drugs or alternative medicine intake, she gave history of intake of around 100 mL of undiluted juice made from fifteen fruits of* Averrhoa bilimbi*, locally known as “Irumban Puli” (Figure 1), three days back, presuming that it would correct her dyslipidemia.

On examination, she was conscious and alert. She had bilateral periorbital puffiness along with pitting pedal edema extending up to one-third of both legs. Her blood pressure was 220/100 mm Hg in the right upper limb in sitting position. Abdominal examination revealed distension of abdomen with abdominal wall edema without evidence of free fluid in abdomen. The rest of clinical examination was within normal limits.

**Figure 2:**
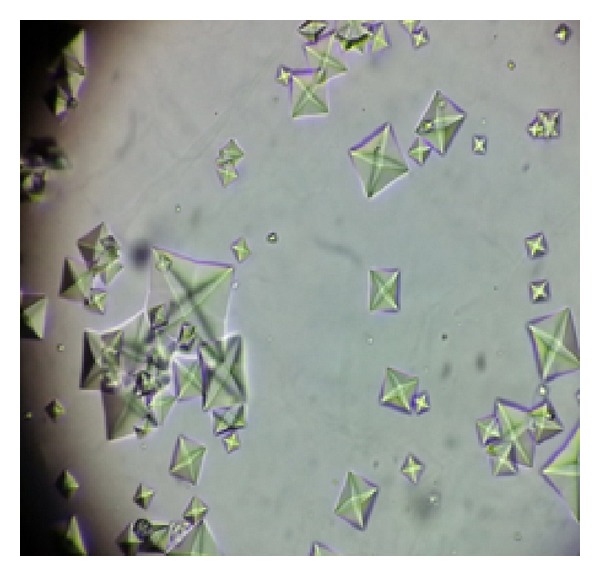
Calcium oxalate crystals in urine in high power view.

**Figure 3:**
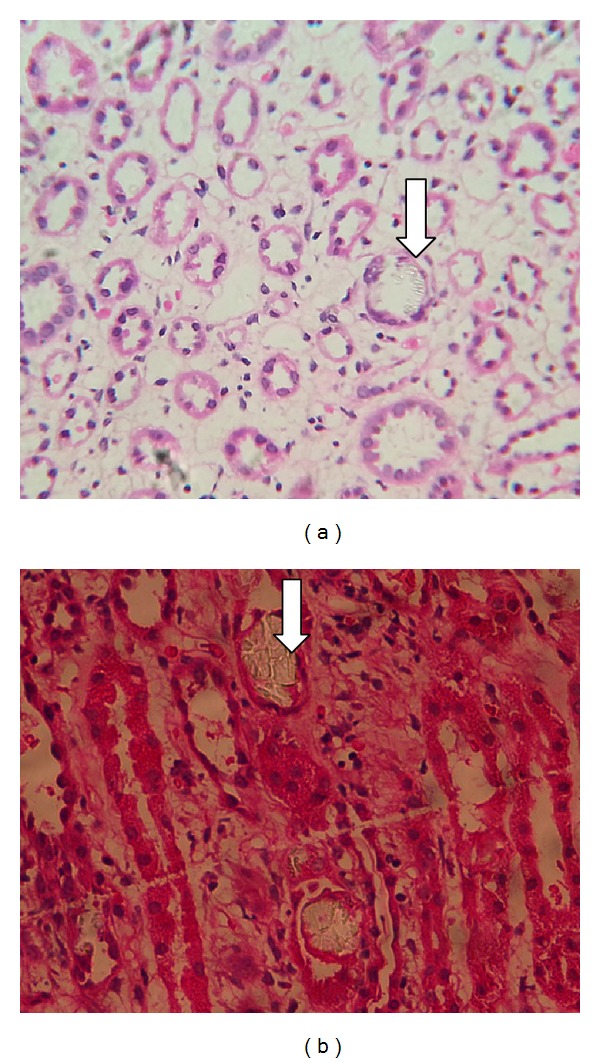
Renal biopsy specimen (40X) with arrow showing calcium oxalate crystals.

Laboratory investigations at time of admission are shown in Table 1. Her urine deposits showed plenty of oxalate crystals (Figure 2). Her 24-hour urinary oxalate done later was negative. Ultrasound abdomen revealed right kidney 10.0 × 5.4 cms and left kidney 10.5 × 4.5 cms with bilateral normal echogenicity and normal corticomedullary differentiation. There was no evidence of calculi or hydronephrosis. A renal biopsy was planned on the initial day of admission but was deferred due to high blood pressure. She was initiated on antihypertensive medications including nitroglycerine infusion. Her blood pressure was controlled on second day and renal biopsy was done which revealed nine glomeruli of which four were obsolescent. The obsolescent glomeruli were small and globally sclerotic. Viable glomeruli were near normal in size and cellularity. Peripheral capillary loops appeared delicate and one glomerulus revealed simplified tuft with wrinkled capillary loops. Tubules showed simplification and vacuolation of epithelium and polarising crystals with fractured glass appearance in the lumen, morphology of which was suggestive of calcium oxalate (Figure 3(a)). Some of the tubules revealed epithelial calcification. There were thin strips of atrophic tubules amounted to around ten % of the cortex. Interstitium showed patchy edema and mild mononuclear infiltrate especially around the atrophic glomeruli and tubules. There was no significant interstitial fibrosis. Large calibre artery showed mild intimal fibroplasia. Interlobular artery revealed medial hypertrophy and arterioles showed hyperplastic change. Immunofluorescence studies were negative for IgG, IgA, IgM, C3, C1q, and both kappa and lambda light chains. A histological diagnosis of acute tubular injury associated with calcium oxalate crystals in a background of mild global glomerulosclerosis and hypertensive vascular changes was made. Patient was managed conservatively as she was nonoliguric. Her renal function initially showed progressive worsening and serum creatinine reached up to 4.9 mg %. She also had high blood pressure which required usage of multiple antihypertensive drugs including amlodipine, atenolol, clonidine, prazosin, telmisartan, minoxidil, and nitroglycerine. Her renal functions gradually improved and blood pressure improved. She was discharged ten days later with serum creatinine of 1.4 mg/dL and three antihypertensive drugs (amlodipine, atenolol, and clonidine).

## 3. Case 2

A forty-seven-year-old male presented with nausea, vomiting, and generalized edema of three-day duration. He had decreased urine output (<400 mL) for past four days. There was no previous history of systemic hypertension or diabetes mellitus. He was diagnosed with dyslipidemia recently. He gave history of ingestion of 150 mL undiluted juice of* Averrhoa bilimbi* for two consecutive days seven days back.

On examination, he had bilateral periorbital puffiness with bilateral pitting pedal edema. Blood pressure was 140/90 mm Hg in the right upper limb in sitting position. Abdominal examination revealed free fluid in abdomen. The rest of clinical examination was within normal limits.

**Figure 4:**
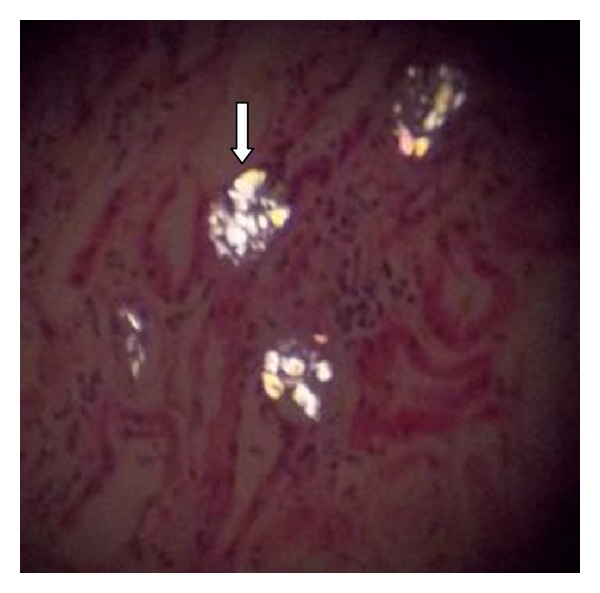
Renal biopsy specimen under polarizing microscope with arrow showing crystals of calcium oxalate.

**Figure 5:**
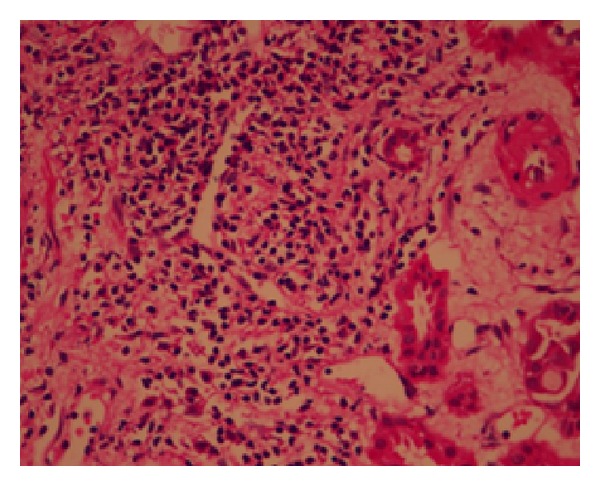
Acute interstitial nephritis.

Laboratory investigations at time of admission are shown in Table 1. Ultrasound abdomen revealed right kidney 10.5 × 5.0 cms and left kidney 10.6 × 4.5 cms with bilateral normal echogenicity and normal corticomedullary differentiation. There were no calculi or hydronephrosis. Renal biopsy done after two sessions of hemodialysis revealed six viable glomeruli with near normal size and cellularity. No endocapillary proliferation or crescents were seen. Tubules revealed marked simplification of epithelium and many refractile polarisable crystals with fractured glass appearance in the tubular lumen (Figures 3(b) and 4). Some of the tubules showed regenerative changes. No tubular atrophy or interstitial fibrosis was seen. Interstitium was edematous with patchy infiltration by lymphocytes, plasma cells, and a few eosinophils (Figure 5). Part of large calibre artery, interlobular artery, and arterioles appeared within normal limits. Immunofluorescence studies showed ten glomeruli with mesangial 1+ IgA and 1+ lambda light chain. IgG, IgM, C3, C1q, and kappa light chains were negative.

As he had severe renal failure with oliguria and features of fluid overload, he was taken for hemodialysis. He required 4 sessions of hemodialysis. In view of dialysis requiring renal failure with histological evidence of acute interstitial nephritis, he was given three daily pulses of methyl prednisolone followed by oral prednisolone. His blood pressure was controlled with amlodipine 5 mg twice daily. His urine output and renal function gradually improved. On discharge after sixteen days, his urine output had improved, blood pressure had normalised, and S. creatinine had decreased to 2.1 mg %. Steroids were tapered and antihypertensive drugs were stopped.

## 4. Discussion

*Averrhoa bilimbi* belongs to the family of Oxalidaceae. It is widely cultivated in the tropics and its origins are not yet clear. Nevertheless, Corrêa reported its presence in India in 1962 [2]. Bilimbi is a small tree 5–10 meters high. Fruits are fairly cylindrical with five broad rounded longitudinal lobes and produced in clusters. Bilimbi fruits are very sour and are commonly used in the production of vinegar, wine, and pickles. It was considered to have medicinal properties and was an ingredient of mixtures against cough, mumps, rheumatism, pimples, and scurvy. The fruit juice has also been used to remove iron-rust stains from clothes and to impart shine to brassware [2].

Both our patients took* Averrhoa bilimbi* juice as a presumed treatment for dyslipidemia. Several studies suggest hypoglycemic, hypolipidemic, antioxidant, and antiatherogenic properties of* Averrhoa bilimbi.* There are reports on hypoglycemic and hypolipidemic effect of ethanolic extract of* Averrhoa bilimbi* leaves in streptozotocin diabetic rats [3]. Bilimbi extract significantly lowered blood glucose by 50% and blood triglyceride by 130% when compared with metformin and distilled water. Bilimbi extract has also been shown to significantly increase the HDL cholesterol concentrations and increases the antiatherogenic index. However, like metformin, bilimbi extract did not affect total cholesterol and LDL cholesterol concentrations, though it significantly reduced the kidney lipid peroxidation levels [3]. Using Triton-induced hypercholesterolemia in rats as a model, bilimbi fruit and its water extract showed remarkable antihypercholesterolemic activity [4]. Oxalic acid content of* Averrhoa bilimbi* fruit has been reported to range between 8.57 and 10.32 mg/g with highest levels seen in half ripe fruit in rainy season and lowest levels in ripe fruits in dry season (25.1 mg/100 g) [2]. The oxalate content of other fruits and vegetables is much less (e.g., cranberry 1.1 mg/100 g, grape 1.6 mg/100 g, tomato 5.5 mg/100 g, pineapple 7.3 mg/100 g, orange 2.2 mg/100 g, apple 0.5 mg/100 g, and banana 3.2 mg/100 g) [5]. Other foods that are rich in oxalate include beans (green and dried), beer, beets, berries, black tea, black pepper, celery, chocolates, cocoa, eggplant, figs (dried), greens (collard green, dandelion green, mustard green, and spinach), green peppers, lemon peel, orange peel, nuts, peanut butter, and okra [6].

There are several case reports of acute oxalate nephropathy due to several agents described in the literature. Bakul et al. had reported a series of cases from five hospitals in the state of Kerala who developed ARF due to acute oxalate nephropathy after consumption of* Averrhoa bilimbi* juice. In that series 7 out of 10 patients had dialysis requiring renal failure after intake of juice but fortunately all had renal recovery [5].

Though* Averrhoa bilimbi* is consumed in several ways, acute kidney injury is mostly seen when it is consumed as raw juice. It is possible that the concentration as well as total amount consumed may have a role to play in the pathogenesis of acute kidney injury. Whether cooking reduces the nephrotoxicity also needs to be studied as there are no reports on acute kidney injury following* Averrhoa bilimbi* pickle intake. Acute oxalate nephropathy has also been reported after ingestion of* Averrhoa carambola* commonly known as star fruit [7], though the oxalic acid content in* Averrhoa carambola* is lesser than* Averrhoa bilimbi* (0.8 to 7.3 mg/g) [2]. There are also case reports of acute oxalate nephropathy following ingestion of ethylene glycol and octreotide. Acute oxalate nephropathy is also described to be associated with chronic pancreatitis and following administration of octreotide and massive doses of ascorbic acid [8–12].

One of our patients had plenty of calcium oxalate crystals in the initial urine sample though her 24-hour urinary oxalate done later was negative. The other patient had no evidence of urinary oxalate excretion which may have been due to decreased excretion in view of severe renal dysfunction. Serum oxalate levels could not be assessed in both our patients as the facility was not available in our hospital. The first patient had very high blood pressure disproportionate to renal dysfunction. A possible toxic effect of* Averrhoa bilimbi* fruit may have contributed to that but this needs further evaluation.

## Figures and Tables

**Table 1:** Laboratory investigations at admission to hospital.

Urine	Case 1	Case 2
Protein	+	Trace
Deposits		
Red blood cells (RBCS)	0-1/hpf	0-1/hpf
White blood cells (WBCS)	5-6/hpf	1-2/hpf
Calcium oxalate crystals	Plenty	Nil
Blood		
Hemoglobin (Hb)	12.3 g%	12 g%
Total white blood cell count (TC)	9000 cells/mm^3^	8300 cells/mm^3^
Differential white blood cell count (DC)	P78 L15 E7	P73 L20 E7
Erythrocyte sedimentation rate (ESR)	17	18
Platelet count	2.7 lakhs/mm^3^	2.18 lakhs/mm^3^
Random blood sugar	70 mg%	106 mg%
Blood urea	74 mg%	142 mg%
Serum creatinine	4.4 mg%	10 mg%
Serum sodium	135 mg%	116 mg%
Serum potassium	4.3 mg%	4.6 mg%
Serum bilirubin total	1.2 mg%	0.7 mg%
Direct	0.8 mg%	0.2 mg%
Indirect	0.4 mg%	0.5 mg%
Serum alanine transaminase (ALT)	22 units	26 units
Serum aspartate transaminase (AST)	34 units	18 units
Total protein	7.5 g%	6 g%
Serum albumin	4.2 g%	4 g%
Serum alkaline phosphatase	69	58
Serum calcium	8.6 mg%	7.7 mg%
Serum phosphorus	4.5 mg%	5.7 mg%
Serum uric acid	9 mg%	10 mg%

## References

[1] Jha V, Parameswaran S (2013). Community-acquired acute kidney injury in tropical countries. *Nature Reviews Nephrology*.

[2] Galvão de lima VLA, de Almeida Mélo E, dos Santos Lima L (2011). Physicochemical characteristics of bilimbi (*Averrhoa bilimbi*). *Revista Brasileira de Fruticultura*.

[3] Pushparaj P, Tan CH, Tan BKH (2000). Effects of *Averrhoa bilimbi* leaf extract on blood glucose and lipids in streptozotocin-diabetic rats. *Journal of Ethnopharmacology*.

[4] Ambili S, Subramoniam A, Nagarajan NS (2009). Studies on the antihyperlipidemic properties of *Averrhoa bilimbi* fruit in rats. *Planta Medica*.

[5] Bakul G, Unni VN, Seethaleksmy NV (2013). Acute oxalate nephropathy due to “*Averrhoa bilimbi*” fruit juice ingestion. *Indian Journal of Nephrology*.

[6] Bushinsky DA, Coe FL, Moe OW (2012). *Brenner and Rectors Diseases of the Kidney*.

[7] Chen C-L, Fang H-C, Chou K-J, Wang J-S, Chung H-M (2001). Acute oxalate nephropathy after ingestion of star fruit. *American Journal of Kidney Diseases*.

[8] Seo JW, Lee J-H, Son IS (2012). Acute oxalate nephropathy caused by ethylene glycol poisoning. *Kidney Research and Clinical Practice*.

[9] Singh A, Sarkar SR, Gaber LW, Perazella MA (2007). Acute oxalate nephropathy associated with orlistat, a gastrointestinal lipase inhibitor. *American Journal of Kidney Diseases*.

[10] Cartery C, Faguer S, Karras A (2011). Oxalate nephropathy associated with chronic pancreatitis. *Clinical Journal of the American Society of Nephrology*.

[11] Gariani K, de Seigneux S, Courbebaisse M, Lévy M, Moll S, Martin P-Y (2012). Oxalate nephropathy induced by octreotide treatment for acromegaly. *Journal of Medical Case Reports*.

[12] Lawton JM, Conway LT, Crosson JT, Smith CL, Abraham PA (1985). Acute oxalate nephropathy after massive ascorbic acid administration. *Archives of Internal Medicine*.

